# Food Variety at 2 Years of Age is Related to Duration of Breastfeeding

**DOI:** 10.3390/nu4101464

**Published:** 2012-10-15

**Authors:** Jane A. Scott, Tsz Ying Chih, Wendy H. Oddy

**Affiliations:** 1 Nutrition and Dietetics, School of Medicine, Flinders University, PO Box 2100, Adelaide 5001, Australia; Email: chih0003@flinders.edu.au; 2 Telethon Institute of Child Health Research, Perth 6008, Australia; Email: wendyo@ichr.uwa.edu.au

**Keywords:** food variety, breastfeeding, toddlers

## Abstract

The aim of this study was to investigate the association of breastfeeding duration and food variety at 2 years of age. A secondary data analysis was undertaken of the Western Australian Pregnancy Cohort (Raine) Study, an ongoing longitudinal study. Data collected from a single 24 h dietary recall of 1905, 2 year-old children were used to calculate two food variety scores; a core food variety score (CFVS) and a fruit and vegetable variety score (FVVS). Multivariate linear regression analysis was used to identify those factors independently associated with the CFVS and FVVS. The mean CFVS was 7.52 (range 1–18) of a possible 34 food items or groups and the mean FVVS was 2.84 (range 0–10) of a possible 16 food items or groups. Breastfeeding duration was independently directly associated with the CFVS (*p* < 0.001) and FVVS (*p* < 0.001). In addition, maternal age was independently directly associated with the CFVS (*p* < 0.001) and FVVS (*p* = 0.001) as was maternal education (CFVS *p* < 0.001 and FVVS *p* = 0.043). The presence of older siblings was independently inversely associated with the CFVS (*p* = 0.003) and FVVS (*p* = 0.001). This study demonstrated a direct modest association between breastfeeding duration and food variety in 2 year-old children, independent of maternal demographic characteristics known to predict food variety in children. This finding supports the hypothesis that flavours transferred in breast milk provide repeated early exposure to different tastes and positively shape children’s food preferences and food variety.

## 1. Introduction

The recommendation to eat a wide variety of nutritious foods is a cornerstone of the Australian Dietary Guidelines [1] and underpins the Australian Guide to Healthy Eating (AGHE) [2]. Eating a varied diet of foods that are biologically diverse or nutritionally distinct from each other is key to achieving adequate coverage of the essential nutrients [1,3]. Amongst children [4] and adults [5] increased food variety is associated with better dietary quality indicators and has been inversely associated with obesity and abdominal adiposity in female adolescents [6] and young adults [7], and risk of colorectal cancer in adults [8].

Infants are born with innate taste preferences, which include a preference for sweet, and probably salty tastes, and the rejection of sour and bitter tastes [9]. These taste predispositions, along with an ability to learn food preferences based on exposure to new foods and flavours [9], will determine a young child’s food preferences and their subsequent eating habits. As food preferences and eating habits form early in life and have been shown to track into later childhood and adulthood [10,11,12], the development of healthy eating habits in early childhood is seen as integral to preventing obesity and diet-related diseases in later life. 

Once complementary feeding commences the acceptance of new foods and flavours usually relies on the repeated exposure to a food or other foods from the same food group [13]. The peak period of accepting vegetables appears to be at the beginning of weaning, but by 2 years of age children are more food neophobic and less willing to try new flavours [14]. There is evidence from studies of both animals and humans that exposure to unique flavours occurs well before the introduction of solid foods with offspring introduced to food flavours in their mothers’ diets via the amniotic fluid during pregnancy and maternal milk after birth [15,16]. 

These early flavour experiences may bias favourably an infant’s acceptance of particular flavours and “program” their later food preferences, and we have previously shown in a separate national study of Australian children that breastfeeding is positively associated with diet quality in later childhood [17]. Therefore the purpose of this study was to further investigate the relationship between breastfeeding duration and food variety amongst a cohort of Australian 2 year-old children.

## 2. Methods

### 2.1. Study Population

Subjects were participants of the longitudinal Western Australian Pregnancy Cohort (Raine) Study which has been described previously [18]. In brief, from 1989 to 1992 pregnant women were recruited from the public antenatal clinics at King Edward Memorial Hospital or nearby private obstetric practices in Perth. The inclusion criteria were that mothers were between 16 and 20 weeks pregnant, had sufficient English proficiency to understand the implications of participation and intended to remain in Western Australia for the foreseeable future to allow follow-up of the child. In all, 2900 women were enrolled and a total of 2804 women (97%) had 2868 live births. These children continue to be followed up at regular intervals and this paper uses data collected during pregnancy and at the one and two year follow-up assessments.

All mothers provided written informed consent, and the study was approved by the Research Ethics Committees of the various participating hospitals and university. Maternal demographic characteristics (age, parity, educational attainment, employment status at 12 months post-partum, living with partner, BMI before pregnancy) were ascertained during pregnancy at 18 and 34 weeks gestation and at years 1 and 2. Demographic data from the 34 week self-completion questionnaire were used primarily for this study. In addition, each mother was given a diary for recording various infant milestones and events, including the time that breastfeeding ceased. When the children reached two years of age their mothers completed a questionnaire related to aspects of their child’s health, which included a 24-h food recall of their child’s food and beverage intake. This questionnaire was checked by the research nurse, with the mother, at the second year clinical assessment.

### 2.2. Development of the Food Variety Score

The AGHE categorizes foods into core and non-core foods. Core foods are described as foods that are essential to eat each day to get enough of the nutrients essential for good health and well-being [2]. The five core food groups are (a) bread, cereals, rice, pasta, noodles; (b) vegetables, legumes; (c) fruit; (d) milk, yoghurt, cheese, and (e) meat, fish, poultry, eggs, nuts, legumes [2]. The AGHE classifies high energy dense/low nutrient dense foods as non-core or “extra” foods. In general, non-core foods are high in fat, salt or sugar content. Foods eaten by the children were broadly categorized into one of these six food groups with each food group having a number of sub-groups to reflect variety within the food group.

To achieve this, individual food and beverage items recorded in the 24-h recall were entered into FoodWorks Professional Version 5 (Xyris Software, Brisbane, 2007), a dietary analysis program which uses the Australian Food and Nutrient database. A complete list of all foods eaten by the infants was generated and food items were then assigned to one of 46 food sub-groups (Table 1). According to the AGHE, legumes can be classified as either a vegetable or a meat substitute. For the purpose of this study, fresh legumes such as green peas were classified as a vegetable whilst dried beans and lentils were classified as a meat substitute.

**Table 1:** Food groups used to calculate food variety scores.

*Milk, dairy*	Cow’s milk ^a^
	Soy milk
	Cheese
	Yogurt ^b^
*Grains and grain products*	Unrefined breakfast cereals ^c^
	Refined breakfast cereals
	Breads and rolls
	Rice and pasta ^d^
	Crackers, pretzels, rice cakes
	Cereal or granola bars
	Pizza/savoury pastry/pies
	Other grains and grain products ^e^
*Vegetables*	Dark green vegetables ^f^
	Deep Yellow/orange vegetables ^g^
	Potatoes ^h^
	French fries/hot chips
	Tomatoes
	Other starchy vegetables ^i^
	Other green vegetables ^j^
	Other vegetables
*Fruits*	Apples
	Bananas
	Berries
	Citrus fruits
	Melon
	Pears
	Stone fruit
	Dried fruit
	Other fruits ^k^
*Meat or other non-dairy protein sources*	Eggs
	Peanut butter, nuts and seeds
	Dried beans and peas, vegetarian meat substitutes
	Red meat ^l^
	Chicken or turkey
	Fish and shellfish
	Hotdogs, sausages, cold cuts ^m^
	Offal and other unspecified meats
*Desserts, sweets, sweetened beverages, and salty snacks (Non-core foods)*	Cakes, pies, cookies, pastries
	Ice cream/frozen yogurt/pudding
	Other desserts ^n^
	Candy
	Chocolate
	Carbonated soft drinks/ sodas
	Fruit-flavoured drinks ^o^
	Salty snacks ^p^
	Added fat ^q^

^a^ Includes flavoured milk; ^b^ Includes dairy yogurt and soy yogurt; ^c^ Includes both ready-to-eat and cooked cereals; ^d^ Where rice or pasta is a primary ingredient; ^e^ Includes rusks and rice cereals; ^f^ Includes broccoli, spinach, and other greens, and romaine lettuce; ^g^ Includes carrots, pumpkin, sweet potatoes, and winter squash; ^h^ Excludes French fries/hot chips; ^i^ Includes corn, green peas; ^j^ Includes leaf, stalk and other green vegetables; ^k^ Includes fruit salad; ^l^ Includes beef, lamb and kangaroo meat, pork; ^m^ Includes ham; ^n^ Includes added sugar, syrups, preserves, milk flavouring and other dessert; ^o^ Includes fruit juice; ^p^ Includes potato chips, popcorn, corn chips, and other types of chips and salty snacks; ^q^ Includes sauces, cream, added fats, spread.

Each food sub-group was categorized into a binary variable, where “0” meant no intake and “1” meant that one or more foods from the food sub-group were consumed. Two food variety scores, a healthy or core food variety score (CFVS) and a fruit and vegetable variety score (FVVS), were calculated by summing the individual food sub-groups. The CFVS included all foods in the five core food groups with the exception of “French fries/hot chips”, “hotdogs and processed meats” and “pizza and savoury pastries” as these are high fat versions of the respective food groups and deemed to be less healthy. Similarly, “French fries/hot chips” were excluded when calculating the FVVS. All food sub-groups were weighted equally and the maximum scores for the CFVS and FVVS were 34 and 16, respectively. 

### 2.3. Statistical Analysis

All data were analysed using SPSS for Windows, Version 19.0 (SPSS Inc., Chicago, IL). Continuous data (CFVS, FVVS, maternal age and breastfeed duration) were assessed for normality by histogram and skewness within −1 to 1. Breastfeeding duration was defined as the age in months that the child stopped breastfeeding and breastfeeding was defined as any breastfeeding, and does not distinguish between exclusive and partial breastfeeding.

A univariate general linear model was used to identify the association between breastfeeding duration and the CFVS and FVVS. Then, nine potential independent variables (maternal age, BMI before pregnancy, parity, employment status, maternal highest education level, living with partner, infant gender, age of introduction of solid foods and presence of older siblings) that might predict food variety were tested by entering them separately into the linear models in addition to breastfeeding duration. Only significant predictor variables were entered into the final multivariate linear models. The alpha level for significance was set at *p* < 0.05. Variables remaining in the models were those that were independently predictive of the food variety score. 

## 3. Results

### 3.1. Characteristics of Participants

Of the 2601 mother-infant pairs that remained in the study at 2 years, 1905 mothers completed a useable 24 h recall, representing 73% of mothers who were contacted at this time point and 68% of those originally recruited who had a live birth. There was no difference in the parity of women who completed or did not complete the 24 h recall but those who completed the 24 h recall were significantly older (28.8 *vs.* 27.0 years, *p* < 0.001) and better educated (76% with post-secondary qualifications *vs.* 70%, *p* < 0.001) than non-completers.

The maternal and infant characteristics of the analysis sample are presented in Table 2. The majority of women were aged 30 years or older, were multiparous, educated beyond secondary school and living with their partner. Just over half of infants were male (52.1%) and the majority had been breastfed beyond 4 months. The mean duration of breastfeeding was 7.7 months (SD ± 7.09) and 181 (10%) of children were never breastfed. The mean score for the CFVS was 7.52 (range of 1–24) and the FVVS was 2.84 (range of 0–10). 

**Table 2:** Maternal, child and family characteristics of subject.

		No. of subjects	Percentage (%)
**Maternal characteristics**			
Age			
	<20	133	7.6
	20–24	215	12.2
	25–29	463	26.3
	30–34	674	38.3
	≥35	276	15.7
Educational attainment			
	Secondary school	885	47.4
	University degree or other ^a^	983	52.6
Employed at 12 months post-partum			
	Yes	610	44%
	No	1202	66%
**Family characteristics **			
Mother living with partner			
	Yes	1591	87.7
	No	223	12.3
Presence of older sibling			
	No	892	47.8
	Yes	976	52.2
**Child characteristics**			
Sex			
	Female	911	47.8
	Male	992	52.1
Breastfeeding duration ^b^			
	Never breastfed	181	10.0
	<1 month	182	10.0
	1–3 months	288	15.8
	4–6 months	306	16.8
	7–9 months	240	13.2
	>9 months	622	34.2

^a^ Other includes trade certificate/apprenticeship, professional registration (non-degree), college diploma/degree, others; ^b^ Age at which child did not receive any breast milk.

### 3.2. Factors Associating with the Food Variety Scores

There were modest but significant direct associations between breastfeeding duration and the CFVS and FVVS, in both the univariate and multivariate linear models (Table 3). Breastfeeding duration remained a significant independent predictor of CFVS and FVVS after adjustment for maternal age, maternal education and presence of older siblings. Maternal age and maternal education were directly associated and the presence of older siblings was inversely associated with both CFVS and FVVS.

**Table 3:** Association of breastfeeding duration^a^ with Core Food Variety Score (CFVS) and Fruit and Vegetable Variety Score (FVVS) at 2-years of age.

		CFVS ^b^			FVVS ^c^		
		Beta	95% CI	p-value	Beta	95% CI	p-value
**Univariate analysis**				****			
Breastfeeding duration		0.066	0.050, 0.082	<0.001	0.038	0.026, 0.050	<0.001
**Multivariate analysis**							
Breastfeeding duration		0.046	0.029, 0.063	<0.001	0.029	0.016, 0.042	<0.001
Maternal age		0.063	0.039, 0.087	<0.001	0.033	0.015, 0.050	<0.001
Maternal educational attainment							
	Secondary school	0			0		
	Uni degree or other ^d^	0.472	0.228, 0.716	<0.001	0.186	0.006, 0.367	0.043
Presence of older sibling							
	No	0			0		
	Yes	−0.371	−0.618, −0.124	0.003	−0.314	−0.496, −0.131	0.001

^a^ Duration of any breastfeeding; ^b^ Core food variety score (CFVS) is the sum of all core food group items excluding French fries/hot chips, pizza and savoury pastries and hotdogs and processed meats, with a maximum score of 34; ^c^ Fruit and Vegetable Variety Score (FVVS) is the sum of all fruits and vegetable items excluding French fries/hot chips; with a maximum score of 16; ^d^ Other includes trade certificate/apprenticeship, professional registration (non-degree), college diploma/degree.

## 4. Discussion

This study demonstrates that breastfeeding duration is directly associated with food variety at two years of age, independent of maternal characteristics known to be associated with diet quality in young children [19,20]. To the best of the authors’ knowledge this is the first study to investigate the association of breastfeeding duration and food variety in toddlers. However, ever having been breastfed has been positively associated with a healthier dietary pattern amongst older Australian children [17]. 

A plausible biological mechanism for these associations can be explained as follows. Children’s food preferences are shaped by repeated exposure of food and flavours pre- and post-natally [16], particularly during the weaning period [9]. Flavours from the mother’s diet are transmitted via amniotic fluid [21] and breast milk [15,22]. Breastfed infants, unlike those who are solely fed formula, are exposed to a variety of flavours well before they are introduced to solid foods. Consequently, breastfeeding is linked with greater acceptability of new food and flavours during the weaning period, a theory which is supported by evidence from animal studies [23,24] and experimental studies in humans [21,25,26,27,28]. 

In this study breastfeeding duration was shown also to be associated with the intake of a greater number of fruits and vegetables. This finding is consistent with the results of a number of studies which have shown breastfed infants to be more accepting of novel fruits and vegetables during the weaning period than formula fed infants [16,29,30]. However, any benefits associated with breastfeeding may be relatively short-lived. Hausner and colleagues [31] demonstrated that while breastfed infants initially had a higher acceptance of a novel flavoured (caraway) puree than formula-fed infants this difference in acceptance disappeared after repeated flavour exposure. Nevertheless, it may take up to 10 repeated exposures to achieve this equalisation [9] and it is unclear whether in non-experimental settings parents of infants will persevere to this extent when introducing new food items into their child’s diets. Breastfeeding may contribute to higher food variety by reducing food neophobia and facilitating the transition to solid foods with lower resistance on the part of the breastfed infant. 

Apart from breastfeeding duration, a number of maternal and family characteristics were associated with children’s food variety. Maternal educational attainment was the strongest independent predictor of the CFVS and also an independent predictor of FVVS. This finding is consistent with the literature which suggests that maternal education is strongly directly associated with children’s diet quality [19,32]. Studies have demonstrated that more educated mothers tend to follow adult healthy eating guidelines and to eat healthier than less educated mothers and that these differences are reflected in the diets of their children [20]. Consistent with the literature, this study also found that food variety, both CFVS and FVVS, amongst 2 years old children was directly associated with maternal age [20] with better food variety observed in children of older mothers.

Another strong determinant of food variety was the presence of older siblings. This study showed that a higher CFVS and FVVS were associated with the absence of older siblings. Robinson and colleagues [20] showed that compared to infants with older siblings, firstborn infants were “more likely to be fed a diet that complied with feeding guidelines and less likely to have a diet characterised by energy-dense, low-micronutrient food”. Similarly, Koh and colleagues [33] reported that infants with older siblings were more likely to have received non-core foods earlier than firstborn infants and a study of English 3 year-olds [19] reported that children with older siblings were more likely to receive a diet comprised primarily of snacks and finger foods and less likely to consume a “traditional” British “meat and two veg” diet. North and colleagues [19] provide two possible explanations for this association. First, mothers who have to attend to the needs of more than one child may not have the time to prepare a traditional family meal and second older siblings may bring “snack” and “junk” foods into the household through for instance, mothers capitulating to the pestering of older children to buy these foods which are highly advertised.

As with all studies this study has a number of limitations. A major limitation was that the CFVS and FVVS were derived from a single 24 h recall that was completed by each infant’s mother. The intake data were of varying quality and for most cases poorly quantified. As a result each variety score reflects exposure to a particular food and not whether an infant had consumed a nutritionally meaningful amount of this food. An assumption is made that food variety is directly related to diet quality but we were unable to validate this assumption in our study. Other studies however, have demonstrated that this assumption in general holds true [4,34]. Furthermore, the analysis sample is not representative of all participants in the Raine study or the general population. Young and less educated women were significantly less likely to complete the 24 h recall than older and better educated women. Both maternal age and education as well as a variety of other socio-demographic variables have been associated with breastfeeding practices [35] and it may be other factors not controlled for in this study that influence both a mother’s decision not to breastfeed and her choice of foods which she feeds her child. Given the modest effect size demonstrated, the possibility of residual confounding cannot be discounted. Another limitation of this study was that few women (<10%) chose not to breastfeed providing a relatively small sample of women to compare against in order to understand the association of breastfeeding and food variety. While the breastfeeding initiation and duration rates reported in this study are not dissimilar to national [36] and local [37] data of the day they are high relative to rates reported for the UK and the USA [38]. It is recommended that the association between breastfeeding duration and food variety and/or diet quality be investigated further in populations with more diverse breastfeeding practices and socio-demographic characteristics. 

Breastfeeding is recognised as the optimal way to nourish infants [39,40] and compared to formula fed infants, breastfed infants have a lower risk of a variety of infections in infancy [39], and obesity, hypertension and raised cholesterol in later life [41,42]. Diet variety is a marker of diet quality [4] which has been associated with obesity among adolescents [6] and young adults [7], and risk of chronic disease such as colorectal cancer in adults [8]. Given our finding that breastfeeding duration is directly associated with diet variety at 2 years of age and that eating patterns have been shown by others to track into later life [10,11,12], this study provides further justification for the promotion and support of breastfeeding.
